# Infant Pneumococcal Carriage During Influenza, RSV, and hMPV Respiratory Illness Within a Maternal Influenza Immunization Trial

**DOI:** 10.1093/infdis/jiz212

**Published:** 2019-05-06

**Authors:** Alastair F Murray, Janet A Englund, Jane Kuypers, James M Tielsch, Joanne Katz, Subarna K Khatry, Steven C Leclerq, Helen Y Chu

**Affiliations:** 1 School of Medicine and Health Sciences, George Washington University, Washington, District of Columbia; 2 Global Health, Milken Institute School of Public Health, George Washington University, Washington, District of Columbia; 3 Seattle Children’s Hospital, Washington; 4 Department of Pediatrics, University of Washington, Seattle; 5 Department of Laboratory Medicine, University of Washington, Seattle; 6 Division of Allergy and Infectious Diseases, University of Washington, Seattle; 7 International Health, Johns Hopkins Bloomberg School of Public Health, Baltimore, Maryland; 8 Nepal Nutrition Intervention Project - Sarlahi, Kathmandu, Nepal

**Keywords:** pneumococcus, influenza, vaccine, RSV, hMPV, maternal immunization

## Abstract

In this post-hoc analysis of midnasal pneumococcal carriage in a community-based, randomized prenatal influenza vaccination trial in Nepal with weekly infant respiratory illness surveillance, 457 of 605 (75.5%) infants with influenza, respiratory syncytial virus (RSV), or human metapneumovirus (hMPV) illness had pneumococcus detected. Pneumococcal carriage did not impact rates of lower respiratory tract disease for these 3 viruses. Influenza-positive infants born to mothers given influenza vaccine had lower pneumococcal carriage rates compared to influenza-positive infants born to mothers receiving placebo (58.1% versus 71.6%, *P* = 0.03). Maternal influenza immunization may impact infant acquisition of pneumococcus during influenza infection.

**Clinical Trials Registration**. NCT01034254.

## Teaser

Among influenza-positive infants, those born to mothers given influenza vaccine had lower pneumococcal carriage rates. Maternal influenza immunization may impact infant pneumococcal acquisition during influenza infection. Pneumococcal carriage did not impact lower respiratory disease rates in RSV, hMPV, or influenza.

While *Streptococcus pneumoniae* is often detected in the nasopharyngeal flora, colonization with this organism is prerequisite to the development of clinically significant invasive pneumococcal disease (IPD). IPD is more likely to occur with initial bacterial acquisition, often occurring in infancy or early childhood [1, 2]. IPD can also occur in the setting of a viral coinfection.

Influenza infection increases the subsequent risk for IPD through inflammation and destruction of airway epithelial cells, neuraminidase-mediated increase in bacterial adherence, and subsequent invasion by *S. pneumoniae* and other bacterial pathogens [3]. In a clinical case-control study of children with severe pneumonia, children with pneumococcal infection were significantly more likely to have evidence of influenza A infection based on serology, highlighting the role of preceding influenza infection in pneumococcal pneumonia [4]. While the effect of pneumococcal carriage on influenza disease has been examined, the occurrence of IPD with other common respiratory viruses, including respiratory syncytial virus (RSV) and human metapneumovirus (hMPV), is not well studied, particularly in developing countries [5].

Two decades of experience with a multivalent pneumococcal conjugate vaccine has shown a substantial reduction in the burden of IPD in infants globally. A time-series analysis of the 7-valent pneumococcal conjugate vaccine in South African children younger than 5 years showed a significant reduction in all pneumonia hospital admissions [6]. In immunized populations, infants too young to receive vaccine, as well as other persons in the population, may benefit from reduced circulation of vaccine serotypes [7].

Inactivated influenza vaccine is often not administered until later in infancy, leaving a window of vulnerability to influenza disease during the first months of life. However, in previously described studies of influenza immunization during pregnancy, protection of infants against influenza via placental transfer of influenza antibody from mother to infant has been well documented [8]. No vaccine intervention is currently available to prevent RSV or hMPV, though monoclonal antibody prophylaxis can be administered in infants at high risk for RSV infection in some countries and maternal RSV vaccine trials are ongoing.

Pneumonia is the most common cause of death in young children worldwide [7]. Our study observed respiratory illness in a birth cohort of infants younger than 6 months in rural Nepal during the period prior to implementation of pneumococcal vaccination in any age group. We studied pneumococcal colonization rates and respiratory illness severity for 3 viruses with a high burden of disease in young infants: influenza, RSV, and hMPV. We further sought to investigate the effect of influenza immunization during pregnancy on pneumococcal carriage in infant influenza illness episodes.

## METHODS

This is a post hoc analysis of a community-based, placebo-controlled, randomized trial of influenza vaccination of pregnant women in a rural district in southern Nepal from April 2011 to May 2014 (Supplementary Figure 1). Pregnant women were enrolled in the second or third trimester and their infants were enrolled at birth with weekly household surveillance for respiratory illness until 180 days of age. Detailed methods and results of the trial have been published [9]. Institutional Review Board (IRB) approval was obtained by Cincinnati Children’s Medical Center, Johns Hopkins Bloomberg School of Public Health (JHBSPH), Institute of Medicine at Tribhuvan University, Kathmandu, and the Nepal Health Research Council. IRBs at Seattle Children’s Hospital, University of Washington, and George Washington University granted oversight to JHBSPH IRB. The maternal influenza trial was registered at clinicaltrials.gov (NCT01034254). On enrollment, pregnant women were randomized to receive either inactivated trivalent influenza vaccine (Vaxigrip, Sanofi Pasteur) or saline placebo [10].

Weekly household-based active surveillance was performed during pregnancy to assess for maternal respiratory illness. A midnasal swab was collected from mothers if they reported fever and an additional clinical symptom (persistent cough, myalgia, sore throat, or nasal congestion) in the past 7 days [8]. Infant surveillance was conducted from birth to 6 months to assess for respiratory illness, defined as fever, cough, difficulty breathing, wheeze, or otorrhea [5]. Disease severity was defined using the 2012 World Health Organization Integrated Management of Childhood Illness criteria [10]. A midnasal swab was collected from infants if mothers reported their infants had experienced any respiratory symptom in the previous 7 days.

Swabs were collected in the field, stored in PrimeStore Molecular Transport Medium (Longhorn Diagnostics LLC, Bethesda, MD), and transported for testing at the University of Washington, Seattle. Previous studies show that the transport media (PrimeStore) lyses and inactivates infectious pathogens while stabilizing RNA and DNA, allowing for safe transport and molecular detection of both viruses and bacteria without immediate refrigeration [9]. Influenza, RSV, and hMPV-positive isolates underwent pneumococcal testing. Respiratory viruses and *S. pneumoniae* were detected using a polymerase chain reaction (PCR) panel including RSV, HMPV, and influenza A and B, and further analyzed using quantitative real time reverse transcription PCR. Pneumococcal DNA extraction was performed with enzymatic lysis and PCR amplification using the LytA-CDC primers and probe.

For infants with repeated viral infections, all viral positives were included in the analysis unless the infant had tested positive for the same virus previously. An a priori multivariable logistic regression was performed to observe factors associated with pneumococcal carriage, with significant variables (*P* ≤.05) selected from univariate logistic regression in Stata 13.0 (StataCorp, College Station, TX). Multivariable logistic regression evaluating pneumococcal carriage by maternal influenza vaccine study arms was adjusted for age. Multivariable logistic regression evaluating presence of clinical pneumonia by pneumococcal carriage was adjusted for preterm birth, low birthweight, and age. Preterm birth was defined as birth <37 weeks completed gestation and low birthweight as birth weight <2500 g, small for gestational age (INTERGROWTH-21st criteria) [11].

## RESULTS

Overall, 53.7% (325/605) of infants in this study with influenza, RSV or hMPV (n = 605) had lower respiratory tract symptoms as compared to 47.8% (n = 1413) of all infants in the larger cohort with any respiratory virus detected (n = 1413/2954). Infants with and without pneumococcal carriage had similar baseline demographics with no significant differences by sex, gestational age, birthweight, or size for gestational age (Table 1 and Supplementary Table 1). Overall mean age at viral illness was 98 days (95% confidence interval [CI], 94–102) with pneumococcal carriage and 78 days (95% CI, 69–86) without carriage (*P* < .001). Households using indoor cookstoves had higher rates of infant pneumococcal carriage (87.1% versus 79.7%, *P* = .018). Infants born June through September (the monsoon season in Nepal) had lower rates of pneumococcal carriage (60.0% versus 69.6%, *P* = .036). However, in this cohort, age at illness was significantly linked to both birth season and indoor cookstove in the household (*P* < .001, *P* = .024, respectively).

**Table 1.** Clinical and Sociodemographic Characteristics of a Birth Cohort of Infants (0*–*6 Months) with Confirmed Influenza Respiratory Infection, Categorized by Maternal Influenza Vaccine Trial Arm and Pneumococcal Carriage

Characteristic	Maternal Vaccine Study Arm, n (%)			Pneumococcal Carriage, n (%)		
	Placebo (n = 102)	Vaccine (n = 70)	*P* Value	*S. pneumoniae* + (n = 113)	*S. pneumoniae* − (n = 59)	*P* Value
Male sex	52 (51)	41 (59)	.33	60 (53)	33 (56)	.72
Jun-Sep birth	40 (39)	34 (49)	.24	39 (35)^a^	35 (60)^a^	.002^a^
						.50
Preterm birth <37 weeks	8 (7.8)	12 (17)	.07	11 (9.7)	9 (15)	.29
Birth weight <2500 g	21 (24)	18 (28)	.41	22 (22)	17 (32)	.18
Small for gestational age	34 (38)	28 (43)	.34	43 (43)	19 (36)	.39
Indoor cookstove	84 (83)	60 (90)	.25	98 (87)	46 (78)	.19
Maternal influenza vaccine intervention	NA	NA	NA	40 (35)^b^	30 (51)^b^	.05^a^
						.03^b^
Age at infection, mean days (95% CI)	96 (87–106)	98 (86–110)	.86	112 (104–120)^b^	68 (55–81)^b^	<.001^a^
						<.001^b^

Missing values in the maternal vaccine study arm were: small for gestational age/low birthweight, 13 (placebo), 6 (vaccine); and indoor cookstove, 1 (placebo), 3 (vaccine).

Missing values in pneumococcal carriage were: small for gestational age/low birthweight, 13 (*S. pneumoniae* +), 6 (*S. pneumoniae* −).

Abbreviations: CI, confidence interval; NA, not applicable; *S. pneumoniae, Streptococcus pneumoniae*.

^a^Significant only on univariate analysis.

^b^Significant on univariate and multivariate analysis.

Of 605 infants with confirmed viral respiratory illness (influenza, RSV, or hMPV), 457 (75.5%) infants had *S. pneumoniae* detected. Evaluating pneumococcal carriage by virus, 113 (65.7%) of 172 infants with influenza, 247 (73.5%) of 336 infants with RSV, and 154 (83.7%) of 184 infants with hMPV had *S. pneumoniae* detected. In the influenza-positive infants, there were no significant differences in sex, gestational age, birthweight, small for gestational age, birth season by maternal receipt of influenza vaccine, or placebo during pregnancy (Table 1).

The mean age at influenza illness with pneumococcal carriage was 112 days (104–120) versus 68 days (55–81) for infants without carriage (*P* < .001, *t* test). The mean age at RSV illness with pneumococcal carriage was 97 days (91–103) versus 73 days (63–82) for infants without carriage (*P* < .001, *t* test). The mean age at hMPV illness with pneumococcal carriage was 101 days (94–109) versus 77 days (57–98) for infants without carriage (*P* = .01, *t* test).

Pneumococcal carriage did not significantly predict reported clinical pneumonia on multivariable logistic regression for influenza (*P* = .07), RSV (*P* = .59), or hMPV (*P* = .80) Supplementary Table 2. Rates of pneumonia in influenza were 38.9% with pneumococcal carriage versus 35.6% without carriage, in RSV 64.4% versus 65.2%, respectively, and in hMPV 52.6% versus 50.0%, respectively.

**Figure 1.**
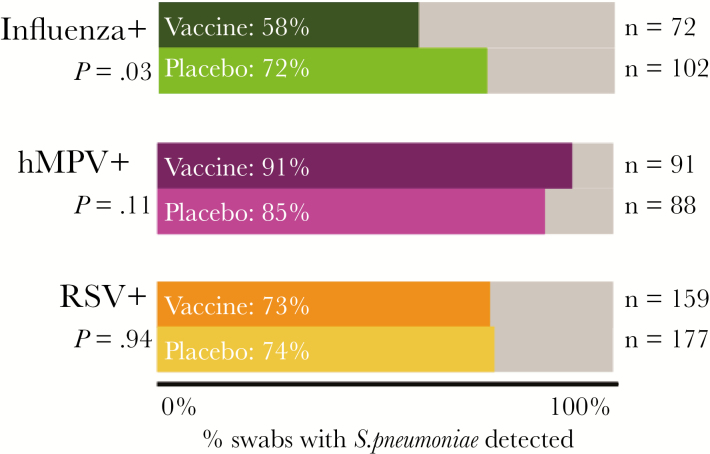
Nasopharyngeal pneumococcal carriage status in infants with respiratory illness in the context of a maternal influenza vaccine trial in Nepal, 2011–2014. Significantly lower rates of pneumococcal carriage were seen in influenza-positive infants of mothers who received the flu vaccine (*P* = .03, age-adjusted multivariable logistic regression). RSV-positive and hMPV-positive infants served as age-adjusted negative control groups. Abbreviations: hMPV, human metapneumovirus; RSV, respiratory syncytial virus; *S. pneumoniae, Streptococcus pneumoniae.*

Considering influenza-positive infants by maternal influenza vaccine status, there was no difference in duration of infant influenza illness (*P* = .61, *t* test) or presence of pneumonia (*P* = .42, multivariable logistic regression). However, observing pneumococcal colonization by maternal influenza vaccine status, influenza-positive infants whose mothers received influenza vaccine were less likely to have *S. pneumoniae* detected than influenza-positive infants whose mothers received placebo (*P* = .03, multivariable logistic regression; Figure 1).

Influenza illness in pregnant women enrolled in the trial was also studied. Among the 75 influenza-positive pregnant women surveyed in this study, 45% (n = 34) had pneumococcal carriage. Of these influenza-positive pregnant women, rates of pneumococcal carriage were 53% (n = 18) among women who received the influenza vaccine versus 40% (n = 16) among women who received placebo (*P* = .32, *Χ*^2^ test).

## DISCUSSION

High rates of pneumococcal carriage were observed with influenza, RSV, and hMPV illness episodes in a birth cohort of infants in rural Nepal prior to the introduction of infant pneumococcal vaccination. Rates of pneumococcal colonization were significantly decreased in influenza-infected infants born to mothers who received influenza vaccine compared to placebo.

Previous studies in this community in Nepal showed 79.4% prevalence of pneumococcal carriage in children ages 1–36 months with higher rates of carriage among healthy controls [12]. A comparable rate of 75.5% pneumococcal carriage in children 0–6 months with confirmed influenza, RSV, or hMPV was observed in this study. The majority of infants had pneumococcus detected at the time of their first observed viral illness. As infants grow older, they are more likely to be colonized with *S. pneumoniae*, likely accounting for the increased age at viral illness in infants with colonization [2].

We found no effect of overall infant pneumococcal carriage on disease severity in viral illness (influenza, RSV, hMPV) in infants followed prospectively during a randomized placebo-controlled trial of maternal influenza immunization. Further, a previous study of repeated respiratory viral infections in this Nepal cohort found no evidence of decreased illness severity or evidence of replacement with another virus after more than one respiratory viral illness [13].

Maternal influenza immunization has been shown to reduce infant influenza acquisition in the first few months of life and protect against adverse birth outcomes, including stillbirth, low birthweight, and preterm delivery in pregnant women exposed to circulating influenza virus in Nepal [5]. A study of maternal influenza immunization in South Africa showed decreased rates of acute lower respiratory illness hospitalizations in infants during the first 3 months of life [14]. The combined effect of maternal influenza immunization and infant pneumococcal vaccination has been shown to further reduce the burden of infant febrile respiratory illness [15].

In our study cohort, significantly lower rates of pneumococcal carriage were observed in influenza-positive infants whose mothers received influenza vaccine during pregnancy as compared to influenza-positive infants whose mothers received placebo. No significant clinical or demographic differences were observed between influenza-positive infants when compared by maternal influenza vaccine study arm (Table 1). This suggests an effect of maternal influenza immunization on the mechanisms impacting infant acquisition of *S. pneumoniae*.

No effect was observed for RSV- or hMPV-positive infants based on maternal influenza vaccine status. Maternal influenza vaccine status also had no effect on rates of clinical pneumonia or duration of illness in influenza-positive infants in this cohort. Rates of pneumococcal carriage among influenza-positive pregnant women who received influenza vaccine were comparable to *S. pneumoniae* carriage rates in influenza-positive women who received placebo.

A study limitation was the lack of collection of samples from asymptomatic infants. It is possible that influenza immunization reduces pneumococcal colonization in infants with and without respiratory symptoms. Additional limitations of this study include the timing of nasal swab collection. Because surveillance was conducted weekly, collection of swabs within a 7-day period could have resulted in variation of swab collection relative to the course of illness [1]. Our study is additionally limited by the potential lower sensitivity of midnasal swabs compared with nasopharyngeal swabs or washes to determine nasopharyngeal bacterial carriage, and the lack of serotyping. Certain pneumococcal serotypes are more likely to be associated with invasive disease and identification of colonizing pneumococcal serotype could further characterize the role of pneumococcal carriage in viral coinfection.

We observed that infant pneumococcal carriage was significantly decreased during active influenza illness in the infants whose mothers received influenza vaccine in pregnancy. In this study cohort, we have previously demonstrated vaccine-derived antibody transfer from mother to infant, but the mechanism of the decreased pneumococcal colonization in these infants may be multifactorial [8]. This finding indicates that maternal influenza immunization may have protective mechanisms affecting infants beyond protection against symptomatic influenza alone.

## Supplementary Data

Supplementary materials are available at *The Journal of Infectious Diseases* online. Consisting of data provided by the authors to benefit the reader, the posted materials are not copyedited and are the sole responsibility of the authors, so questions or comments should be addressed to the corresponding author.

jiz212_suppl_Supplementary_Appendix

## Notes

***Acknowledgment***. We thank all the participants in the trial and the NNIPS staff in Kathmandu and Sarlahi, Nepal and acknowledge the work of Dr. Mark C. Steinhoff, Cincinnati Children’s, Global Health Center.

***Financial support***. This work was supported by National Institutes of Health (grant number K23-AI103105 to H. Y. C.); PATH (H. Y. C. and J. A. E.); and Bill and Melinda Gates Foundation (grant number 50274).

***Potential conflicts of interest***. J. K., H. Y. C., and J. M. T. have received grants from the Bill and Melinda Gates Foundation. J. A. E. has received institutional grants from GlaxoSmithKline, Gilead, Pfizer, Novavax, Chimerix, MedImmune, and Alios. H. Y. C. has received grants from PATH and the Thrasher Foundation, and research funding from Sanofi, GlaxoSmithKline, and Novavax.

All other authors report no potential conflicts. All authors have submitted the ICMJE Form for Disclosure of Potential Conflicts of Interest. Conflicts that the editors consider relevant to the content of the manuscript have been disclosed.
