# Supplementary material for: Infant Pneumococcal Carriage During Influenza, RSV, and hMPV Respiratory Illness Within a Maternal Influenza Immunization Trial
Source: J Infect Dis. 2019 May 6;220(6):956–60. doi: 10.1093/infdis/jiz212 (PMC6688054; doi:10.1093/infdis/jiz212)
Supplement: jiz212_suppl_Supplementary_Appendix [file jiz212_suppl_supplementary_appendix.docx]

**Appendix:**

Diagram illustrating maternal influenza trial enrollment and infant respiratory illness surveillance. Longitudinal household-based weekly surveillance of mothers and infants for respiratory illness was conducted from birth. A mid-nasal swab was collected at the visit if mothers reported their infants had experienced any respiratory symptom reported in the previous 7 days.

**
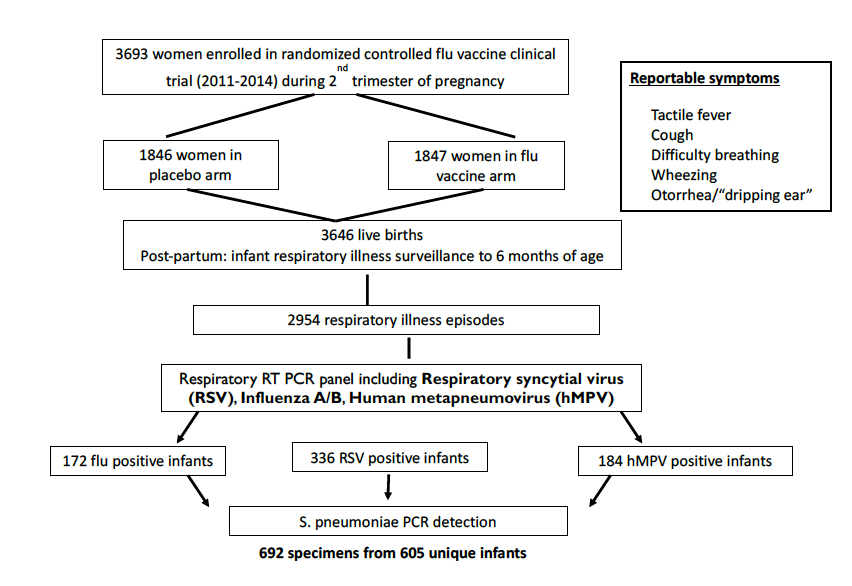
**
